# Preparation of Fe_3_O_4_@ACF Composite Catalytic Electrode and Study of Its Degradation of Antibiotics

**DOI:** 10.3390/nano16070431

**Published:** 2026-03-31

**Authors:** Xuan Liu, Yanqiu Pang, Hanyue Zhang, Yani Liu, Haiyi Yang, Junwei Hou

**Affiliations:** State Key Laboratory of Heavy Oil Processing, China University of Petroleum (Beijing) at Karamay, Karamay 834000, China

**Keywords:** modified activated carbon fiber, heterogeneous electro-Fenton, iron-functionalized cathode

## Abstract

Antibiotics are extensively used in intensive livestock farming for disease prevention, resulting in the discharge of antibiotic-contaminated wastewater into aquatic environments. Addressing this issue, electrocatalytic oxidation has emerged as a promising alternative to conventional chemical oxidation due to its cost-effectiveness and minimal secondary pollution. Central to this technology is the development of catalytic electrodes with high specific surface area and superior electrocatalytic activity. In this work, an Fe_3_O_4_-modified activated carbon fiber electrode (Fe_3_O_4_@ACF) was fabricated via a co-precipitation method. The Fe_3_O_4_@ACF electrode exhibited a hierarchical porous structure with a specific surface area of 940.2 m^2^/g, and demonstrated significantly enhanced oxygen reduction reaction activity with a current density of 21.8 mA·cm^−2^ at –3.25 V vs. Ag/AgCl, which is 2.3 times higher than that of pristine ACF. EIS analysis revealed a low charge transfer resistance of 7.18 Ω, indicating improved electron transfer kinetics. In electro-Fenton degradation of tetracycline, the electrode achieved 82% removal within 120 min with a first-order rate constant of 0.01335 min^−1^, and maintained over 94% of its initial activity after ten cycles. This study offers a viable and sustainable strategy for the efficient treatment of antibiotic-containing medical wastewater.

## 1. Introduction

Antibiotics inhibit the growth and survival of microorganisms and are therefore widely used in healthcare, animal husbandry, and aquaculture [1,2]. Their increasing consumption in recent years has raised concerns about antibiotic residues in aquatic environments and the associated risks to ecosystem sustainability [3]. Tetracycline (TC), a widely used broad-spectrum antibiotic, is often regarded as an emerging contaminant due to its frequent detection and persistence in water bodies [4]. After entering aquatic and soil environments, TC can exert toxic effects on aquatic organisms and microbial communities, disrupt ecological balance, and pose potential risks to human health [5]. Moreover, continuous exposure to TC may promote the emergence and dissemination of antibiotic-resistant bacteria and resistance genes, further aggravating antibiotic resistance and threatening public health [6]. Therefore, developing efficient and sustainable water-treatment technologies to mitigate antibiotic contamination is urgently needed.

Current treatments for emerging organic pollutants include adsorption [7], membrane separation [8], advanced oxidation [9], and biodegradation [10]. Among these, adsorption and advanced oxidation are extensively applied. Adsorption is attractive because of its low cost, simple operation, and rapid pollutant removal [11,12,13]. However, its practical application is limited by poor adsorbent regenerability, high regeneration energy consumption, finite adsorption capacity, and the inherently discontinuous nature of batch replacement/regeneration processes [14,15]. In contrast, advanced oxidation processes (AOPs) are a promising class of technologies capable of mineralizing non-biodegradable organic compounds or converting them into more biodegradable by-products [16,17]. While conventional advanced oxidation processes (AOPs) rely on the external addition of chemical oxidants such as ozone or hydrogen peroxide, electrochemical advanced oxidation processes (EAOPs) generate reactive species in situ via electrode reactions. This enables better process control, reduces chemical transport and storage risks, and enhances environmental compatibility [18]. Electrochemical advanced oxidation processes (EAOPs) have gained increasing attention due to their stable performance, facile controllability, low chemical demand, and environmental compatibility [19,20].

Among EAOPs, electro-Fenton (EF) technology produces H_2_O_2_ in situ at the cathode via electrochemical reduction of dissolved O_2_, while simultaneously enabling continuous regeneration of the Fe^2+^/Fe^3+^ redox catalyst to sustain Fenton reactions [21]. The resulting hydroxyl radicals (∙OH) are highly oxidative and can rapidly and non-selectively degrade refractory organic pollutants into intermediates, which can be further mineralized to CO_2_ and H_2_O [22,23]. In situ H_2_O_2_ generation eliminates the costs and safety concerns associated with transportation and storage [24]. In addition, cyclic Fe^2+^ regeneration can reduce iron-sludge formation and enhance pollutant degradation efficiency [25,26]. For instance, studies have shown that heterogeneous electro-Fenton systems can reduce iron sludge production by over 90% compared to homogeneous Fenton processes, while maintaining degradation efficiencies above 95% [27]. Owing to its robustness against matrix interference, operational simplicity, and high degradation/mineralization capability [28], EF has been widely investigated for the treatment of various refractory organic wastewaters [29,30,31].

To further improve EF performance, extensive efforts have been devoted to developing heterogeneous EF cathodes/catalysts with enhanced electron transfer and H_2_O_2_ utilization. As shown in the Table 1, Cheng et al. [32] fabricated a Fe–Ni LDH@ZIF-67 catalyst-modified carbon-cloth (CC) cathode, achieving 95.6% TC removal within 60 min, compared with 75.7% for Fe–Ni LDH/CC. Under the synergistic action of heterogeneous EF and anodic oxidation, the maximum H_2_O_2_ concentration reached 264 mg∙L^−1^, indicating improved H_2_O_2_ utilization. Cao et al. [33] prepared a Fe_3_O_4_/FeO/Fe_3_C-2-600 catalytic electrode by in situ growth of MIL-88B(Fe) on stainless-steel mesh followed by calcination; the resulting dual-cathode EF system achieved 97.7% TC degradation within 12 min. Cui et al. [34] synthesized Cu/CuFe_2_O_4_ (CCFO) with different CuO ratios using a one-step solvothermal method and obtained 96.3% TC degradation at the optimal composition. Zhang et al. [35] reported CO_2_-activated CuFeC and CuMnC aerogel cathodes that enabled effective treatment of real printing and dyeing wastewater without external addition of Fe^2+^ or H_2_O_2_, with removal efficiencies of 90.5% and 80.3%, respectively, within 60 min. In another study, Fe–Cu/kaolin electrodes were developed for rhodamine B (RhB) degradation in a three-dimensional EF (3D/EF) system, showing RhB removals of 91.6% and 81.1% at initial pH values of 6.71 and 9.0, respectively [36]. Luo et al. [37] investigated TC degradation using Cu-doped Fe@Fe_2_O_3_ core–shell nanoparticles as catalysts and nickel foam as the cathode, achieving 98.1% TC degradation within 2 h and 89.8% mineralization after 6 h. Gao et al. [38] constructed a 3D/EF system using CuFe_2_O_4_ as a heterogeneous catalyst and activated carbon as a granular electrode for semicoke wastewater treatment, achieving 80.9% COD removal under optimal conditions. The synthesis method and resulting morphology of electro-Fenton cathodes play critical roles in determining their catalytic performance. Different synthesis approaches yield distinct morphological features that directly impact key aspects of the electro-Fenton process. Active Site Exposure: Synthesis methods that promote uniform dispersion of catalytically active components maximize the exposure of active sites. Electron Transfer Efficiency: Morphologies that ensure intimate contact between the catalyst and conductive substrate facilitate efficient electron transfer. Mass Transport: Hierarchical pore structures facilitate rapid diffusion of reactants (O_2_, H_2_O_2_) and pollutants to active sites. H_2_O_2_ Generation and Utilization: Morphologies that provide abundant three-phase interfaces (solid catalyst–liquid electrolyte–dissolved O_2_) promote efficient electrochemical reduction of oxygen to H_2_O_2_.

Carbon-based materials can act as a good electron acceptor and transfer channel to improve the conductivity and optimize the adsorption of H- or O-relevant intermediates of g-C_3_N_4_-based catalysts [40,41,42]. Emerging carbon architectures offer additional advantages. For instance, graphdiyne, with its unique sp-/sp^2^-hybridized carbon network and intrinsic bandgap, can modulate interfacial charge transfer more effectively than conventional carbons. Carbon nanocages provide continuous conductive pathways and geometric confinement effects that facilitate both electron transport and mass diffusion, which are critical for gas-involving reactions such as the oxygen evolution reaction (OER) and hydrogen evolution reaction (HER). Ordered mesoporous carbon frameworks, with their well-defined periodic porosity, enable uniform dispersion of g-C_3_N_4_ and maximize accessible active sites while ensuring efficient charge transport [43].

The Fenton reaction is a contact reaction, and the poor electrical conductivity and small specific surface area of FeO or Fe_2_O_3_, which are used as Fenton materials, lead to low Fenton efficiency. Therefore, in this paper, high-performance electro-Fenton catalytic electrodes were prepared by loading Fe_3_O_4_ nanoparticles onto the surface of activated carbon fibers (Fe_3_O_4_@ACF) by chemical precipitation method using carbon nanofibers as substrate.

## 2. Experimental Materials and Methods

### 2.1. Experimental Materials and Reagents

All chemical reagents were analytically pure. Deionized (DI) water was used throughout the experiment. All the reagents used during the experiments, such as hydrochloric acid (HCl, 1 M), activated carbon fiber (ACF, 99.0%), iron sulfate heptahydrate (FeSO_4_·7H_2_O, 99.0%), potassium nitrate (KNO_3_, 99.8%), sodium sulfate (Na_2_SO_4_, 99.0%), sodium hydroxide (NaOH, 98%), and other major analytical reagents, were purchased from the Shanghai Titan Scientific Co., Ltd. (Shanghai, China)

### 2.2. Subsubsection Pre-Processing of ACF

ACF (2 cm × 2 cm, 99.8% purity) was placed in a beaker and ultrasonically cleaned 2–3 times with deionized water to remove residual impurities. Subsequently, a certain amount of 0.1 M HCl solution was added and soaked for 6–8 h to enhance its ability to adsorb ferric ions from solution [44], and finally, filtration was carried out for deionized water washing to remove residual acid and dried at 80 °C to obtain unmodified ACF.

### 2.3. Preparation of ACF-Loaded Fe_3_O_4_ Catalyst Electrode

2.78 g of iron sulfate heptahydrate (FeSO_4_·7H_2_O) and 1.01 g of potassium nitrate (KNO_3_) were dissolved in 60 mL of distilled water, and the reaction was carried out for 20 min with a magnetic stirrer. Subsequently, after vigorous stirring at 60 °C for 20 min, activated carbon fibers were added to the solution, while 6mL of ammonium hydroxide (NH_3_·H_2_O, 28%) was poured into the stirred solution, and the temperature was maintained at 60 °C (1 h). After the reaction, it was taken out and left to stand for 24 h, then washed with deionized water and dried at 80 °C in a preheated vacuum drying oven to obtain modified activated carbon fiber (Fe_3_O_4_@ACF).

The pretreated pure ACF and optimized Fe_3_O_4_@ACF prepared by the co-precipitation method were stored in a sealed bag for subsequent experiments.

### 2.4. Analytical Instrument

The surface morphology and elemental composition of the samples were characterized using a Hitachi SU4800 scanning electron microscope (SEM) (manufacturer: Hitachi High-Tech Corporation, Minato-ku, Tokyo, Japan) equipped with an energy dispersive X-ray spectrometer (HORIBA, 7593-H) (manufacturer: HORIBA, Ltd., Kyoto, Japan). Energy-dispersive X-ray spectroscopy (EDS) was used in conjunction with SEM to obtain characteristic spectra for qualitative and quantitative elemental analysis. The structure and crystal phase composition of the samples were investigated using an Ultimo IV X-ray diffractometer (CuKα radiation) (manufacturer: Thermo Fisher Scientific, Madison, Wisconsin, United States of America). The samples were analyzed by X-ray photoelectron spectroscopy (XPS) for surface composition and chemical valence states using a Kratos AXIS SUPRA XPS (manufacturer: Kratos Analytical Ltd., Manchester, United Kingdom). The samples were analyzed using a Micromeritics ASAP 2460 fully automated specific surface area and porosity analyzer (N_2_ desorption) (manufacturer: Micromeritics Instrument Corporation, Norcross, Georgia, United States of America). The elemental distribution on the sample surface was characterized by transmission electron microscopy (TEM) mapping analysis using a JEOL JEM 2100F system (200 kV voltage) (manufacturer: JEOL Ltd., Akishima, Tokyo, Japan).

### 2.5. Electrochemical Performance Study

Electrochemical tests, including linear scanning voltammetry (LSV) and electrochemical impedance spectroscopy (EIS) were carried out using Shanghai Chenhua CHI660E electrochemical workstation. These tests were carried out in 0.1 M Na_2_SO_4_ aqueous solution using a three-electrode system, in which the cathode material prepared in this study was used as the working electrode, a platinum sheet was used as the counter electrode, and an Ag/AgCl electrode was used as the reference electrode.

Linear Scanning Voltammetry (LSV): LSV has a test voltage range of –4.0 V to 2.0 V and a scan rate of 50 mV/S. Electrochemical Impedance Spectroscopy (EIS): EIS measurements are performed with an amplitude of 5 mV and a frequency range of 1 × 10^6^ to 1 × 10^−2^ Hz.

### 2.6. Study of Oxidative Degradation Properties of Electrofenton

The experiments were carried out in a 250 mL vessel. A platinum sheet electrode was used as the anode for the reaction, while the Fe_3_O_4_@ACF material was used as the functionalized cathode (both measuring 2 cm × 2 cm). Subsequently, 100 mL of 20 mg/L TC solution was added to the vessel with 0.05 M Na_2_SO_4_ as the supporting electrolyte. Throughout the Electro-Fenton reaction, the solution was stirred and homogenized using a magnetic stirrer, and the current was supplied by an adjustable constant voltage DC power supply. Then air was passed into the solution at a flow rate of 200 mL/min to establish a non-homogeneous Electro-Fenton oxidation system. Ten minutes before the start of the experiment, a mechanical stirrer was activated for rapid mixing and air was introduced to form an air-saturated Electro-Fenton system. The constant voltage DC power supply was then started by connecting the positive and negative terminals to the poles of the power supply.

The tetracycline solution was scanned at full wavelength with a UV-visible spectrophotometer to determine its maximum absorption wavelength. Subsequently, the absorbance at the maximum wavelength was detected and monitored using a UV-visible spectrophotometer. The UV-visible degradation efficiency was calculated as:

Removal rate (%) = (C_0_ − C_t_)/C_0_ × 100% (1)

where C_0_ is the initial concentration of tetracycline (mg/L); C_t_ is the concentration of tetracycline at t min (mg/L); K is the quasi-primary rate constant for tetracycline degradation; and T is the reaction time (min).

### 2.7. Mechanistic Analysis of the Electro-Fenton System

First, 3 mM TBHP, IPA and MeOH were used as the bursting agents, which were added to the Electro-Fenton oxidation system, and the concentration of tetracycline was monitored by UV-Vis spectrophotometer. Subsequently, free radicals that play an important role in tetracycline degradation were investigated using electron paramagnetic resonance spectroscopy (EPR) on a Bruker EMX PLUS instrument in Germany in the range of 3460–3560 G. The results of this study are summarized in the following table.

## 3. Results and Discussion

### 3.1. Microstructure and Composition

**Figure 1:**
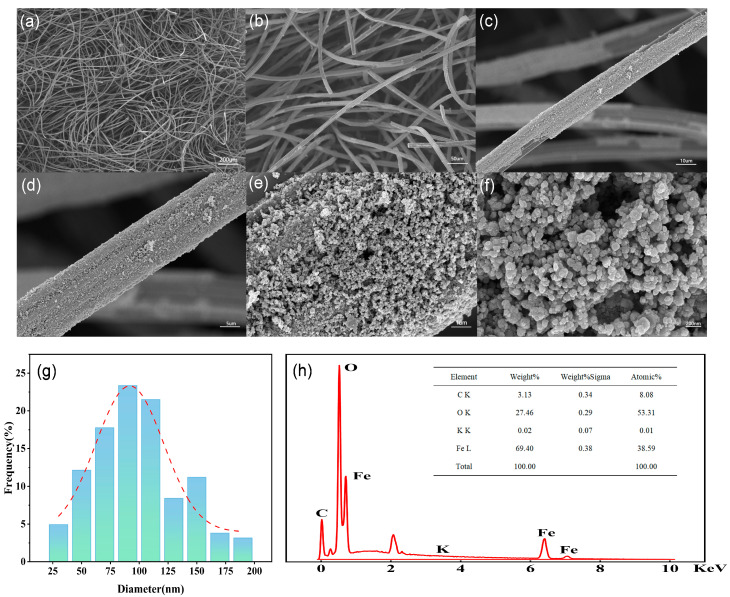
SEM images of Fe_3_O_4_@ACF at different magnifications (**a**–**f**) and Particle size distribution histogram of Fe_3_O_4_@ACF (**g**); EDS of Fe_3_O_4_@ACF (**h**).

Figure 1 shows SEM images and EDS energy spectra of the prepared Fe_3_O_4_@ACF, as well as quantitative data on the distribution of each element. In Figure 1a–c, the samples show a large number of dense ACF filaments with a diameter of 11 μm. The ACF exhibits a unique, interlaced rod-like fibrous structure with many grooves on the surface. This unique morphology contributes to the large specific surface area and abundant active sites of the material. Figure 1d–f shows increased surface roughness after Fe_3_O_4_ loading. In addition, the aggregation of smaller Fe_3_O_4_@ACF particles to form larger-sized clusters of particles stacked in a disordered manner, as well as a layer of Fe_3_O_4_ flocs loading, resulted in the roughness and inhomogeneity of the microstructure of the sample surface. The EDS spectrum (Figure 1h) shows uniform distribution of Fe, O, C, and K elements in the composite, with weight percentages of 69.40% (C), 27.46% (O), 3.13% (Fe), and 0.02% (K). From the above results, it is shown that Fe_3_O_4_ particles have been successfully loaded onto the ACF surface.

**Figure 2:**
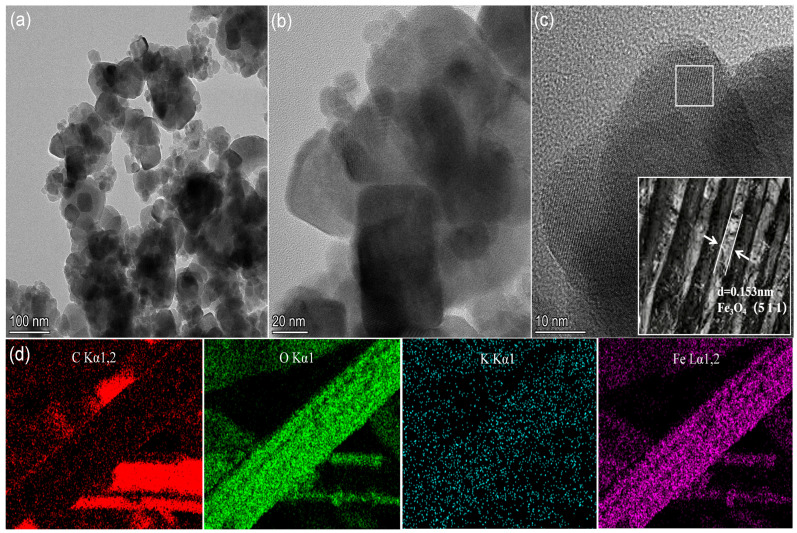
TEM (**a**,**b**) and HRTEM (**c**) images of Fe_3_O_4_@ACF, and elemental mapping image of Fe_3_O_4_@ACF (**d**).

The microscopic morphology of Fe_3_O_4_@ACF was observed by transmission electron microscopy, as shown in Figure 2a,b. The composite particles are close to spherical and loaded on the ACF in a cluster shape, and it can be observed that a clear boundary appears between the Fe_3_O_4_ crystals and the ACF material, indicating that Fe_3_O_4_ is tightly adhered to the ACF surface. The high-resolution transmission electron microscopy (HRTEM) image (Figure 2c) shows that the lattice spacing of the as-prepared Fe_3_O_4_ lattice is 0.153 nm and corresponds to the iron tetraoxide (511) crystal plane. In addition, the EDS elemental mapping in Figure 2d confirms the presence of C, O, Fe and K elements in Fe_3_O_4_@ACF, where C, O and Fe are very uniformly distributed and K is sporadically dispersed. It further indicates that the Fe_3_O_4_@ACF non-homogeneous phase-like Fenton catalyst has been successfully constructed.

**Figure 3:**
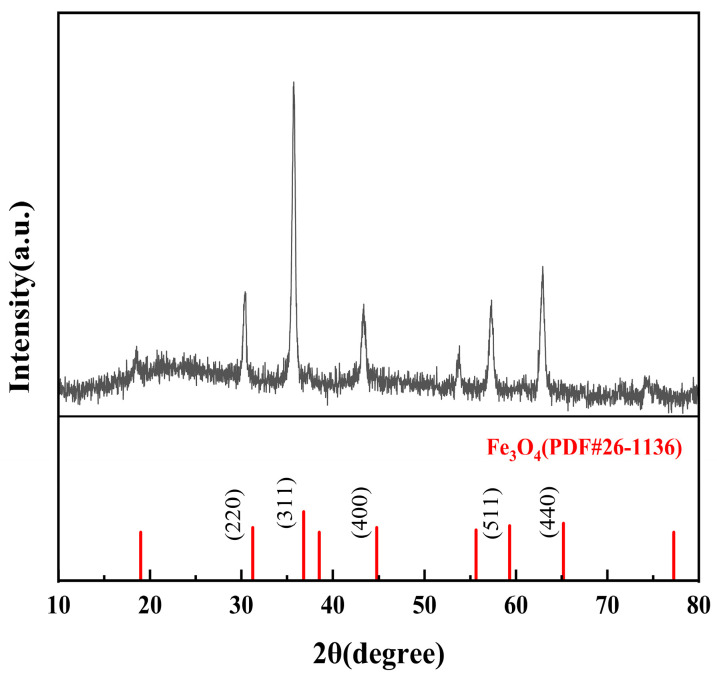
X-ray powder diffraction pattern of Fe_3_O_4_@ACF.

**Figure 4:**
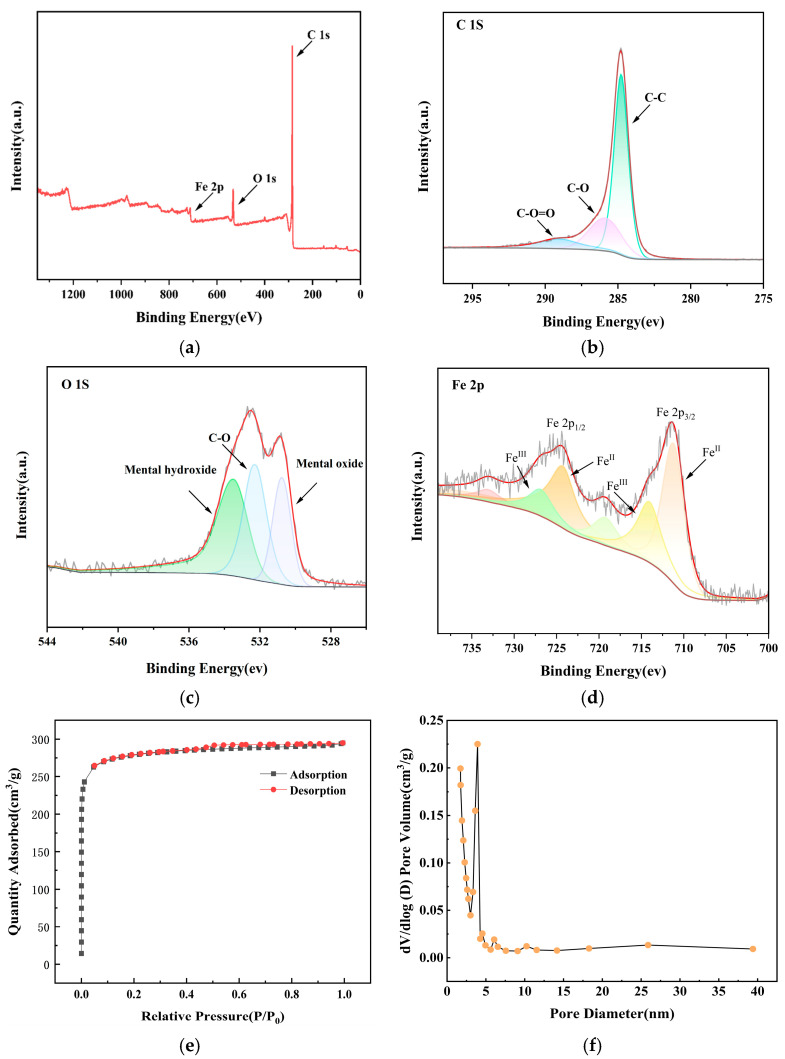
Total XPS spectrum of Fe_3_O_4_@ACF (**a**); C 1s peak (**b**); O 1s peak (**c**); Fe 2p peak (**d**); Nitrogen adsorption–desorption isotherm of Fe_3_O_4_@ACF (**e**) and corresponding pore size distribution curve (**f**).

The XRD pattern of the Fe_3_O_4_@ACF composite (Figure 3) exhibits diffraction peaks that can be indexed to the spinel structure of magnetite (Fe_3_O_4_, JCPDS No. 26-1136). Slight shifts to lower 2θ values are attributed to nanoscale particle size (~100 nm) and partial surface oxidation to maghemite (γ-Fe_2_O_3_), as confirmed by XPS (Figure 4d). No detectable impurity phases such as α-Fe_2_O_3_ or FeO are observed. The coexistence of Fe_3_O_4_ and surface γ-Fe_2_O_3_ is consistent with the literature on air-exposed magnetite nanoparticles and does not compromise the catalytic activity, as both phases are active in Fenton-like reactions. This result provides direct evidence for the successful crystallization of Fe_3_O_4_ on the ACF substrate. Concurrently, the absence of any sharp diffraction features attributable to activated carbon indicates that the ACF matrix retains its amorphous structure within the composite. The HRTEM image in Figure 2c reveals clear lattice fringes with an interplanar spacing of 0.153 nm, which corresponds to the (511) plane of Fe_3_O_4_. This assignment is consistent with the distinct XRD peak observed at 59.303° (2θ) indexed to the (511) reflection (JCPDS No. 26-1136). Furthermore, the XRD pattern in Figure 3 exhibits additional peaks corresponding to the (220), (311), (400), and (440) planes, confirming the phase purity of the Fe_3_O_4_ nanoparticles.

The chemical states and surface composition of the Fe_3_O_4_@ACF composite were further elucidated by XPS. As presented in Figure 4a,d, the high-resolution C 1s spectrum (Figure 4b) was deconvoluted into three peaks at 284.1 eV (C=C/C–C), 285.0 eV (C–O), and 290.9 eV (O=C–O). The presence of C–O species is corroborated by the O 1s spectrum (Figure 4c), which exhibits three components at 531.61 eV (C–O), 529.70 eV (metal oxides), and 532.83 eV (metal hydroxides). The metal hydroxide is Fe(OH)_3_ and it is possible that some Fe(OH)_3_ did not undergo dehydration to form Fe_3_O_4_ due to incomplete drying at the sampling location. The metal oxides signal originates from a mixed phase of Fe_3_O_4_ and γ-Fe_2_O_3_. This is confirmed by the Fe 2p spectrum (Figure 4d), where the Fe 2p_3_/_2_ and Fe 2p_1_/_2_ core-level peaks are centered at 711.2 eV and 724.9 eV, respectively, matching the standard values for Fe_3_O_4_ [45]. The detailed fitting reveals contributions from both Fe^2+^ (peaks at ~710.8 eV and ~724.3 eV) and Fe^3+^ (peaks at ~712.9 eV and ~726.27 eV) oxidation states. Moreover, the distinct satellite peak near 718.7 eV is characteristic of Fe^3+^ in γ-Fe_2_O_3_ [46], indicating partial surface oxidation of the Fe_3_O_4_ nanoparticles. Collectively, the XRD and XPS analyses provide consistent and conclusive evidence for the successful loading of Fe_3_O_4_ onto the ACF surface.

The specific surface area and pore architecture of the samples were characterized through N_2_ adsorption–desorption measurements, with the corresponding isotherms and pore size distribution profiles presented in Figure 4e–f [47]. The obtained isotherms exhibit a pronounced hysteresis loop within the relative pressure (P/P_0_) range of 0.4 to 1.0. This feature is diagnostic of mesoporous materials, arising from the capillary condensation and evaporation of nitrogen within pores of specific geometry. According to the IUPAC classification, the observed pattern aligns with a Type II isotherm displaying an H3-type hysteresis loop. This classification is characteristic of aggregated plate-like particles, which generate slit-shaped or wedge-shaped pores. The complexity of the pore network, particularly the presence of such non-uniform slit pores, accounts for the noted divergence between the adsorption and desorption branches and the hysteresis loop morphology. Quantitative analysis of the desorption branch via the BJH method revealed a bimodal pore size distribution, confirming the coexistence of micropores (centered around 1.7–2.0 nm) and mesopores (in the range of 2.0–5.0 nm), indicative of a hierarchical pore structure within the Fe_3_O_4_@ACF composite. The morphological characteristics of the Fe_3_O_4_@ACF composite are intrinsically linked to its electrochemical degradation performance. The interlaced fibrous ACF network provides a high surface area that enables uniform dispersion of Fe_3_O_4_ nanoparticles (~100 nm), preventing aggregation and maximizing exposure of active Fe sites. The tight adhesion between Fe_3_O_4_ and the conductive ACF substrate facilitates efficient electron transfer, as evidenced by the reduced charge transfer resistance (7.18 Ω) and enhanced ORR activity. Furthermore, the hierarchical pore structure (micropores of 1.7–2.0 nm and mesopores of 2.0–5.0 nm) synergistically promotes pollutant adsorption and rapid mass transport of reactants, ensuring efficient utilization of electrogenerated reactive oxygen species.

### 3.2. Electrochemical Performance Analysis

**Figure 5:**
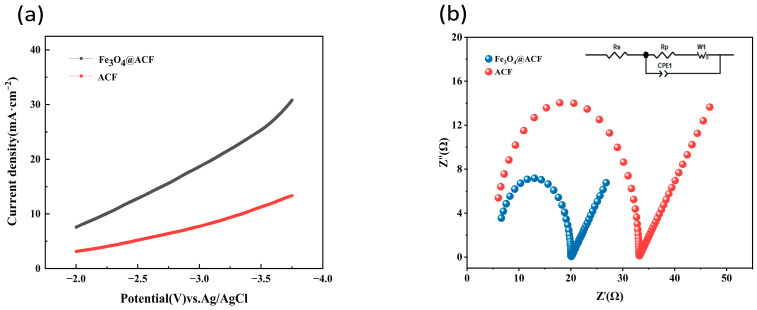
Linear sweep voltammetry curves (**a**) and electrochemical impedance spectra (**b**) of pure ACF and Fe_3_O_4_@ACF.

The electrocatalytic performance of the materials was first assessed through linear sweep voltammetry (LSV). As depicted in Figure 5a, the Fe_3_O_4_@ACF composite exhibits significantly enhanced oxygen reduction reaction (ORR) activity compared to pristine ACF. At −3.25 V vs. Ag/AgCl, the current density reaches 21.8 mA·cm^−2^ for Fe_3_O_4_@ACF, which is 2.3 times higher than that of pure ACF (9.4 mA·cm^−2^). This improvement indicates that Fe_3_O_4_ nanoparticles effectively promote the electrocatalytic process.

To elucidate the origin of this enhanced activity, electrochemical impedance spectroscopy (EIS) was employed to probe the charge transfer kinetics [48] at the electrode-electrolyte interface (Figure 5b). Nyquist plots show distinct semicircles in the high-frequency region, corresponding to charge transfer resistance (Rct). Quantitative fitting shows that Fe_3_O_4_@ACF possesses a substantially lower Rct value (7.18 Ω) compared to pure ACF, indicating significantly facilitated electron transfer. In the low-frequency region, the linear Warburg component reflects mass transport limitations, with Fe_3_O_4_@ACF exhibiting a shallower slope, suggesting improved diffusion kinetics. The EIS fitting results demonstrate that the charge transfer resistance (Rct = 13.02 Ω) of Fe_3_O_4_@ACF is significantly lower than that of pure ACF (Rct = 27.35 Ω), indicating a marked reduction in electronic transfer impedance (Table S1). This facilitates electron transfer during reactions and lowers electrochemical resistance, thereby enhancing power density and electron flux between acceptors and donors. These findings confirm that Fe_3_O_4_ loading effectively improves the material’s electron transport efficiency. The similarity in CPE1 parameters suggests negligible differences in interfacial capacitance characteristics, indicating that the loading process did not compromise the material’s interfacial structural stability.

The synergistic combination of LSV and EIS analyses provides compelling evidence for the superior electrocatalytic performance of Fe_3_O_4_@ACF. The substantially increased ORR current density directly demonstrates enhanced catalytic activity, while the reduced charge transfer resistance reveals more efficient interfacial electron transfer processes. These complementary findings collectively confirm that Fe_3_O_4_ modification effectively optimizes both the catalytic sites and charge transport pathways in the ACF-based electrode.

### 3.3. Catalytic Degradation Properties of Tetracycline by Fe_3_O_4_@ACF

To evaluate the versatility of the composite electrode, degradation experiments were conducted on tetracycline (TC) solutions. The effects of several parameters on degradation efficiency were systematically investigated, including the degradation method, electrode material, current density, pH value, electrode spacing, solution temperature, TC concentration, and electrode area.

**Figure 6:**
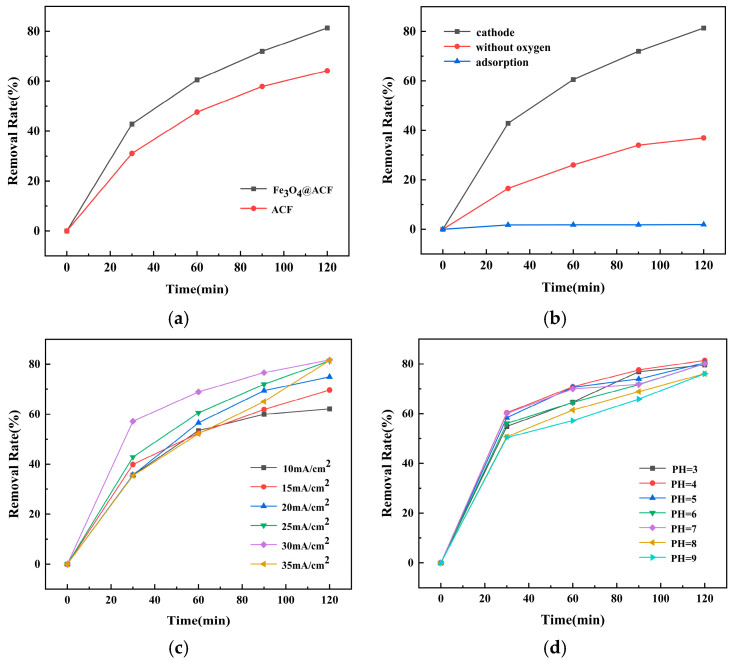

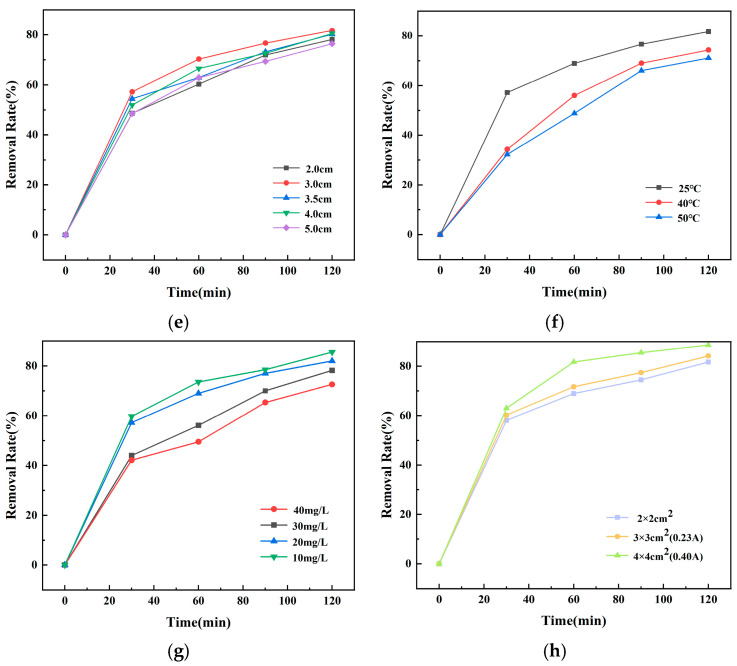
Comparison of Electro-Fenton oxidation on the degradation of pure ACF and Fe_3_O_4_@ACF (**a**); Comparison of different degradation methods (adsorption, anaerobic, cathodic) (**b**); Effect of current density on Electro-Fenton oxidation degradation (**c**); Effect of initial pH on Electro-Fenton oxidation degradation (**d**); Effect of electrode plate spacing on Electro-Fenton oxidation degradation (**e**); Effect of solution temperature on Electro-Fenton oxidation degradation (**f**);Effect of TC concentration on Electro-Fenton oxidation and degradation (**g**); Effect of electrode area on Electro-Fenton oxidation and degradation (**h**).

To elucidate the mechanism by which the Fe_3_O_4_@ACF composite electrode degrades tetracycline via the electro-Fenton process, a series of control experiments were performed (Figure 6b). The results demonstrate that the degradation efficiency was substantially suppressed under both adsorption-only and anaerobic conditions. In the adsorption-only test, the adsorption of tetracycline on ACF was below 2%, indicating a negligible contribution of physical adsorption to the overall removal. This minimal adsorption is likely attributable to the near-saturation of the ACF support during the material-preparation stage. Under oxygen-deficient operation, the interruption of continuous oxygen supply to the cathode severely hindered the oxygen-reduction reaction and the consequent electrogeneration of H_2_O_2_, leading to a pronounced decline in degradation. Nevertheless, a measurable residual degradation activity persisted, which can be explained by two concurrent pathways: oxygen produced at the anode via the oxygen-evolution reaction may diffuse to the cathode and be reduced to H_2_O_2_, while a small fraction of water molecules at the anode may be directly oxidized to yield ·OH radicals, both contributing to limited pollutant degradation in the absence of external aeration. Taken together, these findings confirm that the efficient degradation of tetracycline by Fe_3_O_4_@ACF is predominantly governed by the electro-Fenton catalytic oxidation when the composite acts as the cathode, rather than by physical adsorption or direct anodic oxidation pathways.

The comparison of Electro-Fenton oxidation performance of ACF and Fe_3_O_4_@ACF under the same conditions is shown in Figure 6a. The removal rate of ACF at 120 min was only 64.21%, while Fe_3_O_4_@ACF exhibited a removal rate of 82% at 120 min. This phenomenon may be attributed to the fact that the iron particles in Fe_3_O_4_@ACF act as a bridge for electron transfer, which polarizes the cathode and improves electron transfer efficiency [49]. This observation is consistent with the electrochemical properties of the material. Therefore, in the Electro-Fenton reaction, the Fe_3_O_4_@ACF composite electrode, exhibiting superior oxygen reduction reaction (ORR) electrocatalytic activity, was able to produce more H_2_O_2_. This, in turn, reacted with the iron active sites on the catalyst surface to generate more reactive species, ultimately enhancing the TC removal rate.

Figure 6c shows the effect of different current densities (10, 15, 20, 25, 30, and 35 mA·cm^−2^) on TC degradation. It can be seen that the highest removal rate (82%) was achieved at a current density of 30 mA·cm^−2^. This enhancement is attributed to iron particles acting as electron transfer bridges, polarizing the cathode and improving electron transfer efficiency, consistent with electrochemical properties. However, excessive current densities can promote side reactions [24], such as water electrolysis, which reduces the removal efficiency.

The effect of the initial solution pH on TC removal was investigated. Initial pH values in the range of 3–7 resulted in over 80% removal at 120 min. TC removal slightly decreased with increasing initial pH, consistent with the optimal pH for ·OH generation in the Fenton system of approximately 4.0 [50]. At low pH, the scavenging effect of H^+^ is enhanced, leading to a decrease in the oxidizing capacity of the Fenton process [51]. The formation of HO_2_^−^ at higher pH may lead to a decrease in H_2_O_2_ yield [52]. In fact, the removal of TC by Fe_3_O_4_@ACF at pH 9 still reached more than 75% at 120 min. Therefore, Fe_3_O_4_@ACF shows superior activity in Electro-Fenton systems under acidic, neutral, and even weakly alkaline conditions, expanding the pH applicability in practical applications.

Figure 6e shows the effect of electrode spacing on the oxidation performance of the Electro-Fenton process [53]. It can be seen that when the electrode spacing is 3 cm, the removal rate reaches 81.72% at 120 min. Deviations above or below this value result in a decrease in removal efficiency. Excessive spacing reduces the rates of electromigration and diffusive mass transfer during electrochemical reactions [54]. When the spacing is too small, concentration polarization occurs, resulting in fewer organic molecules being free to migrate, which decreases mass transfer efficiency and thus affects the treatment efficiency of the Electro-Fenton system [26].

Figure 6f shows the effect of temperature on the performance of the Electro-Fenton process. It can be seen that the degradation efficiency decreases with increasing temperature, but the final degradation rate remains above 70%. At higher temperatures, the solubility of oxygen in water decreases, leading to reduced H_2_O_2_ production via electrocatalysis. In addition, at elevated temperatures, some H_2_O_2_ may decompose directly into H_2_O, significantly reducing the amount of ·OH available to degrade the contaminant and thus lowering the removal rate. Therefore, the Electro-Fenton system is not suitable for the direct treatment of high-temperature wastewater, and cooling the wastewater prior to the Electro-Fenton reaction is necessary to achieve better removal efficiency.

To investigate the effect of initial TC concentration on the catalytic performance of Fe_3_O_4_@ACF, TC concentrations of 10, 20, 30, and 40 mg/L were tested, as shown in Figure 6g. The catalytic degradation efficiency of the Fe_3_O_4_@ACF Electro-Fenton system decreased with increasing TC concentration. This inhibition can be attributed to the heterogeneous catalytic process at the interface of the liquid phase (TC solution) and solid phase (Fe_3_O_4_@ACF) [55]. The presence of a high TC concentration leads to competitive adsorption of TC and H_2_O_2_ on the Fe_3_O_4_@ACF catalyst surface. As a result, it becomes difficult for H_2_O_2_ to compete with TC molecules because the higher TC concentration limits the adsorption and subsequent degradation of H_2_O_2_. In addition, the decomposed TC intermediates occupy the active sites of the catalyst, which further inhibits the adsorption of H_2_O_2_ and leads to a decrease in degradation efficiency.

Figure 6h shows the effect of different electrode areas on the electrocatalytic performance. It can be seen that the degradation efficiency gradually increases with increasing electrode area, providing preliminary evidence of the potential of the composite electrodes for future industrial-scale applications.

### 3.4. Reusability and Practicality of Fe_3_O_4_@ACF

**Figure 7:**
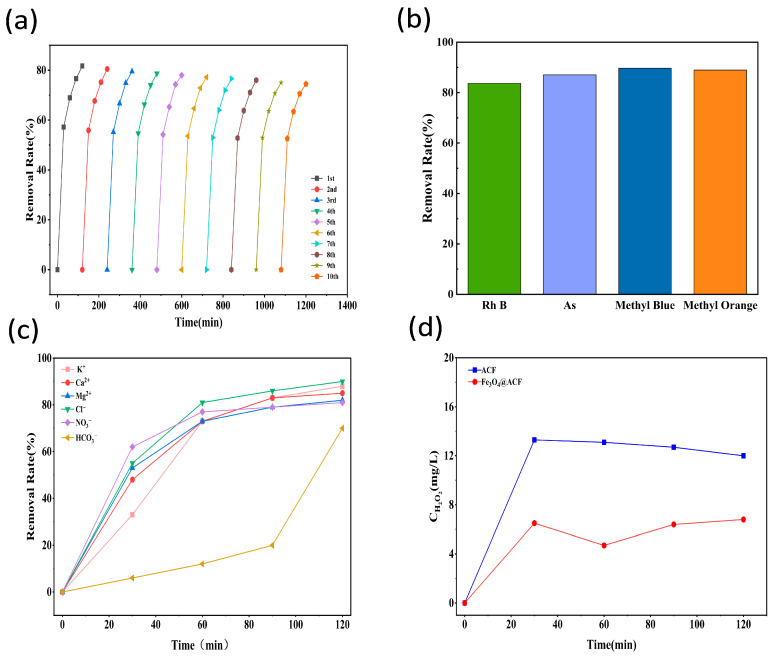
Stability of Fe_3_O_4_@ACF electrodes (**a**) and degradation experiments of different organic compounds (**b**); Degradation experiments for interfering ions (K^+^, Ca^2+^, Mg^2+^, Cl^−^, NO^3−^, and HCO^3−^) experiments (**c**); Time-dependent H_2_O_2_ concentration curves generated by pristine ACF and Fe_3_O_4_@ACF electrodes in the electro-Fenton system (**d**).

In addition to degradation efficiency, electrode stability is also important from a practical application perspective. Therefore, five consecutive degradation cycles were performed to evaluate the reusability of the Fe_3_O_4_@ACF electrode. As shown in Figure 7a, the TC degradation rate was maintained at around 75% and did not decrease significantly over ten cycles. This indicates that the Fe_3_O_4_@ACF electrode is stable and reusable, and this nanomaterial shows great promise for sustainable use and recycling in Electro-Fenton systems.

In addition, the performance of the Fe_3_O_4_@ACF composite electrode was evaluated by degrading other difficult-to-degrade pollutants, including Rhodamine B (RhB), arsenic-containing solutions (As), Methyl Blue, and Methyl Orange. As shown in Figure 7b, the removal efficiencies of these pollutants reached 83.64%, 87.06%, 89.72%, and 88.95%, respectively, within 120 min. The results indicate that the Fe_3_O_4_@ACF composite electrode has broad applicability for the degradation of various pollutants, highlighting its potential for treating a wide range of organic contaminants. we have further supplemented the anti-interference experiments against common coexisting ions in actual industrial wastewater, and the results show that our system maintains stable degradation performance in the presence of 100 mg/L of common cations (K^+^, Ca^2+^, Mg^2+^) and anions (Cl^−^, NO_3_^−^, HCO_3_^−^), with only the bicarbonate ion showing a significant inhibitory effect, which provides solid data support for the practical application potential of the system in complex water matrices. The results of Figure 7d reveal that the pristine ACF electrode achieves a peak H_2_O_2_ concentration of 13.3 mg/L at 30 min, which gradually decreases to 12.0 mg/L by 120 min. In contrast, the Fe_3_O_4_@ACF electrode exhibits a lower peak H_2_O_2_ concentration (6.5 mg/L at 30 min), followed by a slight recovery to 6.8 mg/L at 120 min. This observation aligns with the enhanced electro-Fenton catalytic activity of Fe_3_O_4_@ACF: the Fe_3_O_4_ nanoparticles act as efficient Fenton catalysts, promoting the rapid decomposition of generated H_2_O_2_ into highly reactive ·OH radicals, which accelerates pollutant degradation and results in a lower steady-state H_2_O_2_ concentration compared to ACF. This provides direct experimental evidence linking Fe_3_O_4_ modification to improved utilization of H_2_O_2_ for pollutant mineralization, supporting our mechanistic hypothesis of enhanced ORR-derived electro-Fenton activity.

### 3.5. Analysis of the Main Controlling Factors of the Fenton Reaction

**Figure 8:**
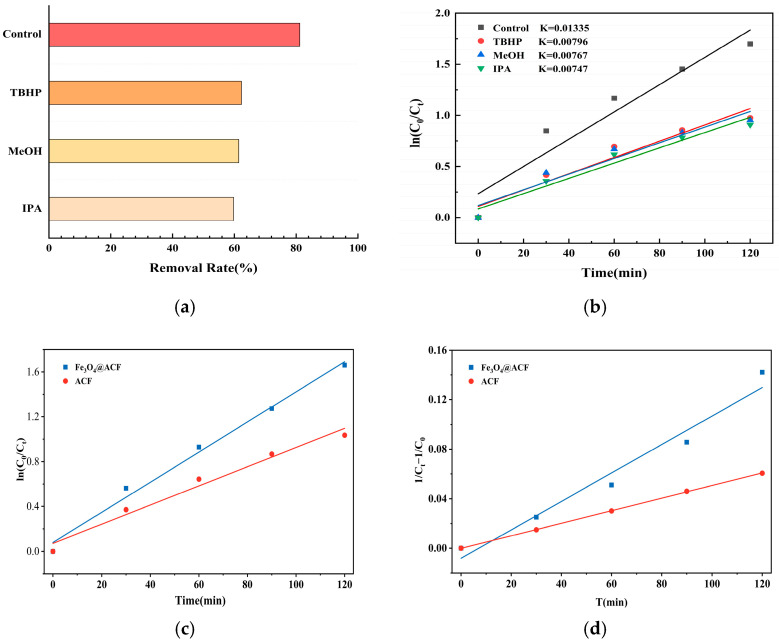

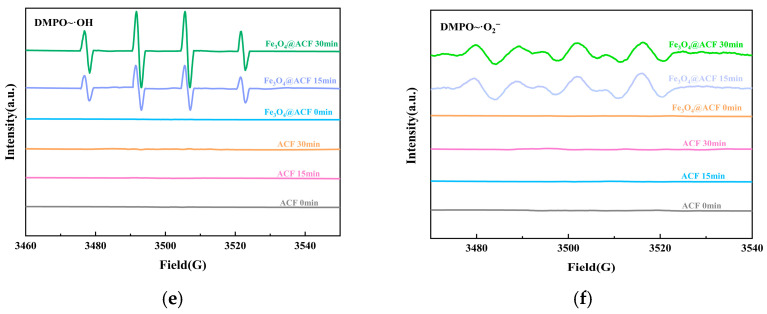
Effect of quenching agent on TC removal rate at 120 min (**a**); Kinetic analysis of the quenching group (**b**); pseudo-first-order (**c**) and pseudo-second-order (**d**) kinetic fitting plots for tetracycline hydrochloride degradation over Fe_3_O_4_@ACF and pristine ACF electrodes; Spin-capture EPR spectra of DMPO~∙OH in a methanol dispersion system for Fe_3_O_4_@ACF and ACF (**e**); Spin-capture EPR spectra of DMPO~∙O_2_^−^ in a methanol dispersion system for Fe_3_O_4_@ACF and ACF (**f**).

The mechanism of TC degradation was investigated by quencher and EPR experiments. The reactive species ∙OH and ∙O_2_^−^ produced during degradation were quenched by 3 mM MeOH (or TBHP) and IPA. In Figure 8a, the removal efficiency of TC decreased from 82% to 61.44% and 62.3% when MeOH and TBHP were added, respectively. When IPA was added, the removal efficiency was 59.75% after 120 min. The contributions of ·OH and ·O_2_^−^ to the removal process were calculated as 20.56% (or 19.7%) and 22.25%, respectively. To better study the kinetics of the TC degradation process, a first-order kinetic model was proposed as follows:

ln(C_0_/C_t_) = kt (2)

where C_0_ is the initial concentration of tetracycline (mg/L); C_t_ is the concentration of tetracycline at t min (mg/L); k is the quasi-primary rate constant for tetracycline degradation; and t is the reaction time (min).

As shown in Figure 8b, the first-order pseudo-kinetic constants k for each reaction after addition of quencher were calculated kinetically as 0.01335 (min^−1^), 0.00796 (min^−1^), 0.00767 (min^−1^), and 0.00747 (min^−1^), respectively. In summary, the addition of MeOH, TBHP and IPA significantly reduced the reaction rate, which corresponded to the decrease in the removal efficiency. This confirms that ∙OH and ∙O_2_^−^ play a major role in the degradation of TC.

This conclusion was further verified by EPR experiments. Samples were taken at 0 min, 15 min and 30 min of the energization reaction using pure-ACF and Fe_3_O_4_@ACF electrodes to detect the signal intensity of DMPO~∙OH and DMPO~∙O_2_^−^. As shown in Figure 8c,d, at 0 min, no ∙OH and ∙O_2_^−^ were produced by the catalytic reaction of both electrodes, and the quantitatively determined concentrations of ∙OH and ∙O_2_^−^ signals were 0. After 15 min of the reaction, DMPO~∙OH and DMPO~∙O_2_^−^ signals were detected for the Fe_3_O_4_@ACF electrode. Figure 8c shows the characteristic peaks of DMPO~∙OH with intensity ratio of 1:2:2:1. Figure 8d shows the signals with intensity ratio of 1:1:1:1, which is in agreement with the peaks of DMPO~∙O_2_^−^. This finding suggests that the presence of Fe_3_O_4_ significantly enhances the catalytic ability of pure ACF in the Fenton reaction. Thus, the Fe_3_O_4_@ACF composite electrode produced ∙OH and ∙O_2_^−^ in the catalytic reaction, confirming that the loading of Fe_3_O_4_ promoted the production of ∙OH and ∙O_2_^−^. The results showed that the doping of Fe_3_O_4_ effectively improved the efficiency of the Electro-Fenton reaction. The quenching and EPR experiments fully demonstrated that ∙OH and ∙O_2_^−^ are the main radicals in the reaction process.

Cathode:


O_2_ +2H^+^ + 2e^−^ → H_2_O_2_ (3)

Fe^2+^ + H_2_O_2_ → Fe^3+^ + ·OH + OH^−^ (4)

Fe^3+^ + H_2_O_2_ → Fe^2+^ + ·O_2_^−^ + 2H^+^ (5)

·OH/·O_2_^−^ + organic pollutants → intermediate → CO_2_ + H_2_O (6)

In the Electro-Fenton reaction, the supplied oxygen is converted into H_2_O_2_, (Equation (3)) following the classical two-electron oxygen reduction reaction (ORR) pathway [56]. This process involves the redox cycling between Fe^2+^ and Fe^3+^, which leads to the generation of ∙OH and ∙O_2_^−^ (Equations (4) and (5)). Subsequently, the generated ∙OH or ∙O_2_^−^ attack the organic pollutants, producing intermediates that are eventually mineralized into CO_2_ and H_2_O (Equation (6)). Loaded Fe_3_O_4_ particles act as electron transfer bridges, enhancing electron transport, reactive oxygen species generation, and degradation efficiency.

## 4. Conclusions

In this study, a Fe_3_O_4_@ACF composite cathode was successfully fabricated via an optimized co-precipitation method. The Fe_3_O_4_ nanoparticles (~100 nm) were uniformly loaded onto the ACF support, as confirmed by SEM, TEM, XRD, and XPS analyses. The Fe_3_O_4_@ACF electrode exhibited superior electrochemical performance, achieving a 2.3-fold higher ORR current density than pristine ACF and a low charge transfer resistance of 7.18 Ω. When employed as a cathode in an electro-Fenton system, it achieved 82% tetracycline degradation within 120 min under optimal conditions (30 mA·cm^−2^, 3 cm electrode spacing, room temperature), following first-order kinetics with a rate constant of 0.01335 min^−1^. The electrode demonstrated excellent stability, with less than 3% performance decay over five cycles, and broad applicability, achieving >80% removal for various organic pollutants. Mechanistic studies confirmed that ·OH and ·O_2_^−^ are the dominant reactive species, with Fe_3_O_4_ nanoparticles serving as electron-transfer mediators to enhance ROS generation. Overall, the Fe_3_O_4_@ACF composite offers a facile, stable, and efficient cathode material with broad pH applicability, presenting a promising solution for the treatment of antibiotic-contaminated wastewater.

## Figures and Tables

**Table 1:** Previously reported iron-based cathode materials for Electro-Fenton degradation of organic pollutants in wastewater.

Electrode Material	Organic Dye	Synthesis Method	Morphology	Concentration (mg/L)	Removal Efficiency (Time)	Current Density/ Voltage	Ref.
Fe-Ni LDH@ZIF-67	Tetracycline (TC)	In situ growth	Nanosheet array	10	95.6% (60 min)	100 mA∙cm^−2^	[32]
Fe_3_O_4_/Fe0/Fe_3_C-2-600	Tetracycline (TC)	MOF-derived calcination	Nanoparticles	10	97.7% (12 min)	3.5 mA∙cm^−2^	[33]
3,6-Cu/CuFe_2_O_4_/CB@GF	Tetracycline (TC)	Solvothermal	Nanoclusters	50	96.3 ± 1.8% (3120 min)	30 mA∙cm^−2^	[34]
CuFeC	2,4,6-trichlorophenol (2,4,6-TCP)	Pyrolysis	Porous	40	90.5% (60 min)	7 mA∙cm^−2^	[35]
Fe-Cu/kaolin	RhodamineB (Rh B)	Impregnation-calcination	Particle electrodes	20	97.2% (60 min)	10 V	[36]
Cu-doped Fe@Fe_2_O_3_ (CFF)	Tetracycline (TC)	Solvothermal	Core–shell nanoparticles	20	98.1% (3120 min) 90.0% (5120 min) 72.2% (7120 min)	40 mA∙cm^−2^	[37]
CuFe_2_O_4_	200 mL semi-coking water	Solvothermal	Nanoparticles	\	COD:80.9%, 120 min	4 V	[38]
FeCuC	Methylene (MB)	Sol–gel	Porous	50	98% (30 min) 99% (60 min)	10 mA (4.1 V)	[39]
FeCuC	Real dyeing waste water	Sol–gel	Porous	\	TOC:64% (30 min) 83% (60 min)	10 mA (4.1 V)	[39]

## Data Availability

The original contributions presented in the study are included in the article, further inquiries can be directed to the corresponding authors.

## Supplementary Materials

The following supporting information can be downloaded at: https://www.mdpi.com/article/10.3390/nano16070431/s1, Figure S1: Schematic diagram of electrode preparation; Figure S2: Pseudo-first-order (a) and pseudo-second-order (b) kinetic fitting plots for tetracycline hydrochloride degradation over Fe_3_O_4_@ACF and pristine ACF electrodes; Figure S3: TOC removal rates of tetracycline hydrochloride over pristine ACF and Fe_3_O_4_@ACF electrodes as a function of reaction time; Figure S4: Catalytic tetracycline degradation mechanism diagram using ACF-loaded Fe_3_O_4;_ Figure S5: Current-time curve of Fe3O4@ACF at 8 V constant voltage; Table S1: Parameters fitted to the EIS test; Table S2: Previously reported materials for Electro-Fenton degradation of TC [57,58,59,60,61]; Table S3: Specific surface area, pore volume and average pore diameter parameters of pristine ACF and Fe_3_O_4_@ACF samples.
