# Supplementary material for: Preparation of Fe3O4@ACF Composite Catalytic Electrode and Study of Its Degradation of Antibiotics
Source: Nanomaterials (Basel). 2026 Mar 31;16(7):431. doi: 10.3390/nano16070431 (PMC13074807; doi:10.3390/nano16070431)
Supplement: Supplementary file 1 [file nanomaterials-16-00431-s001.zip › nanomaterials-4223920-supplementary.pdf]

Supplementary Materials

Preparation of Fe_3O_4@ACF Composite Catalytic Electrode and Study of Its Degradation of Antibiotics

Xuan Liu, Yanqiu Pang, Hanyue Zhang, Yani Liu, Haiyi Yang and Junwei Hou *

State Key Laboratory of Heavy Oil Processing, China University of Petroleum (Beijing) at Karamay; Karamay 834000, China
* Correspondence: junweihou@cupk.edu.cn

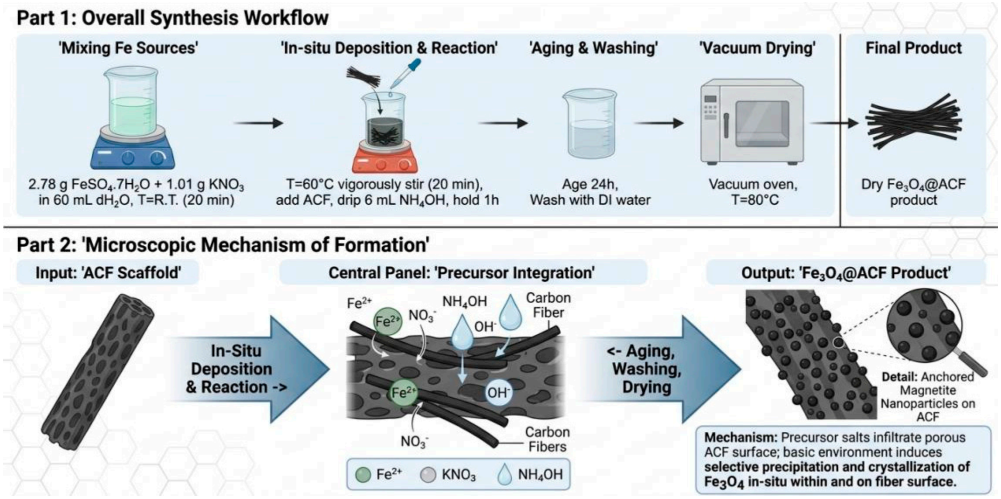

Figure S1. Schematic diagram of electrode preparation

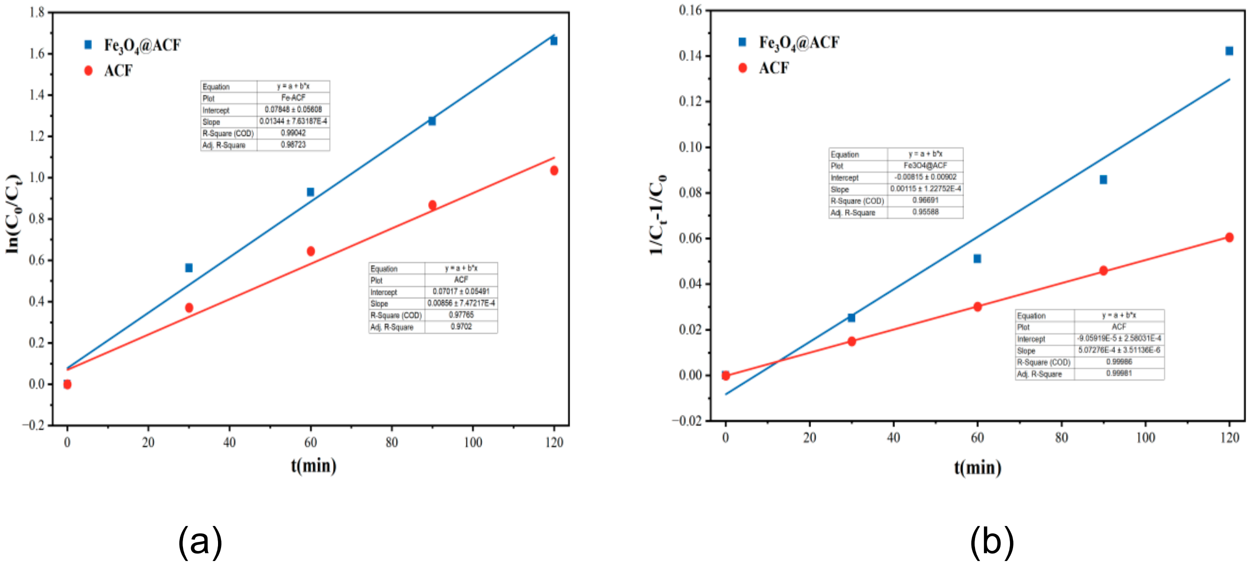

Figure S2. Pseudo-first-order (a) and pseudo-second-order (b) kinetic fitting plots for tetracycline hydrochloride degradation over Fe_3O_4@ACF and pristine ACF electrodes.

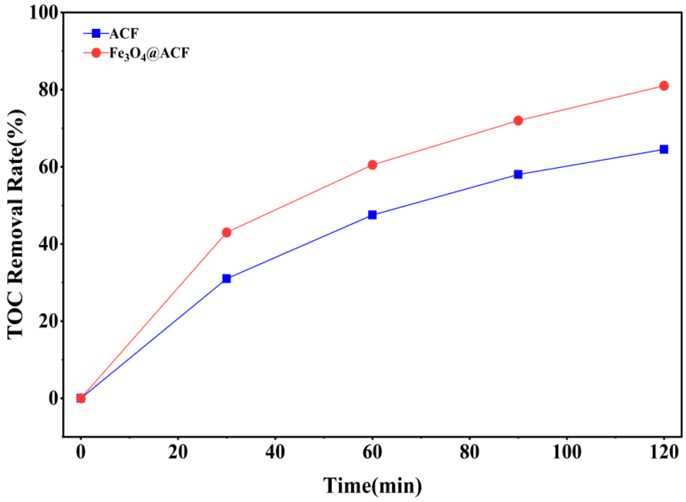

Figure S3. TOC removal rates of tetracycline hydrochloride over pristine ACF and Fe_3O_4@ACF electrodes as a function of reaction time.

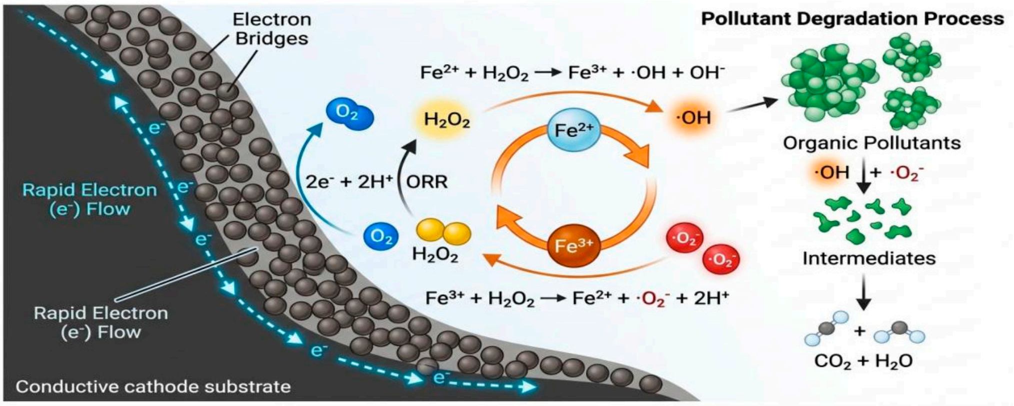

Figure S4. Catalytic tetracycline degradation mechanism diagram using ACF-loaded Fe_3O_4

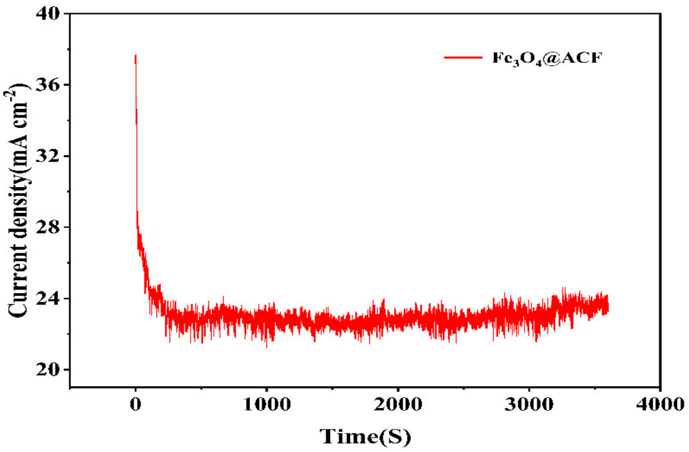

Figure S5. Current-time curve of Fe_3O_4@ACF at 8 V constant voltage

Table S1. Parameters fitted to the EIS test

Sample	Rs（Ω）	Rct（Ω）	CPE1	
			Y0	n1
ACF	4.12	27.35	4.57×10^{-8}	0.96
Fe_3O_4@ACF	5.02	13.02	6.18×10^{-8}	0.96

Table S2. Previously reported materials for Electro-Fenton degradation of TC

Electrode material	Organic dye	Concentration(mg/L)	Removal efficiency (time)	Current density voltage	Ref
BDD	Tetracycline (TC)	10	65%(30 min)	0.5 A cm^{-2}	[0]
FeMn-LDH/g-C_3N_4	Tetracycline (TC)	50	75.9% (120 min)	30 mA cm^{-2}	[0]
Ti/SnO_2-Sb	Tetracycline (TC)	100	55%(120 min)	5 mA cm^{-2}	[0]
IrO_2 + PDS	Tetracycline (TC)	50	75.84% (180 min)	30 mA cm^{-2}	[0]
Ti/Ta_2O_5-IrO_2	Tetracycline (TC)	20	78% (30 min)	10 mA cm^{-2}	[0]

Table S3. Specific surface area, pore volume and average pore diameter parameters of pristine ACF and Fe_3O_4@ACF samples.

Sample	Specific Surface Area (m^2/g)	Pore Volume (cm^3/g)	Average Pore Diameter (nm)
Pristine ACF	1099.86	0.4564	1.66
Fe_3O_4@ACF	940.23	0.4028	1.71

References

57. Chen, T.S.; Tsai, R.W.; Chen, Y.S.; et al. Electrochemical Degradation of Tetracycline on BDD in Aqueous Solutions. *Int. J. Electrochem. Sci.* **2014**, *9*, 8422–8434.

58. Qi, J.; Li, M.; Yin, E.; et al. Degradation of tetracycline under a wide pH range in a heterogeneous photo bio-electro-fenton system using FeMn-LDH/g-C_3N_4 cathode: Performance and mechanism. *J. Environ. Manag.* **2024**, *360*, 121111.

59. Zhi, D.; Qin, J.; Zhou, H.; et al. Removal of tetracycline by electrochemical oxidation using a Ti/SnO_2–Sb anode: Characterization, kinetics, and degradation pathway. *J. Appl. Electrochem.* **2017**, *47*, 1313–1322.

60. Li, H.; Zhang, J.; Du, Y.; et al. Electrochemical activation of peroxydisulfate using an IrO_2 electrode for the efficient degradation of acid orange 74: Mechanisms of different activation methods. *J. Water Process Eng.* **2023**, *55*, 104208.

61. Dong, H.; Chi, W.; Gao, A.; et al. Electrochemical Degradation of Tetracycline Using a Ti/Ta_2O_5-IrO_2 Anode: Performance, Kinetics, and Degradation Mechanism. *Materials* **2021**, *14*, 4325.
